# Immune Cell Dynamics in Cardiac Diseases: Insights From Single‐Cell Sequencing

**DOI:** 10.1111/jcmm.70846

**Published:** 2025-10-26

**Authors:** Weirong Zeng, Jiao Li, Weiwei Liu, Bei Shi, Ranzun Zhao, Yan Wang

**Affiliations:** ^1^ Department of Cardiology Affiliated Hospital of Zunyi Medical University Zunyi China

**Keywords:** cardiac diseases, immune cell, single‐cell sequencing

## Abstract

Immune cell‐mediated immune‐inflammatory responses play a key role in the development of cardiac diseases. To study cardiac health and the underlying pathophysiological mechanisms, researchers must thoroughly understand immune cell functions and characteristics. However, the complex cellular heterogeneity and inter‐cellular interactions remain largely unexplored. Single‐cell sequencing, a high‐throughput technique for sequencing genomes and transcriptomes at the individual cell level, not only enables the discovery of new cell types but also provides a detailed assessment of cellular heterogeneity and reveals each cell's unique contribution to disease. This technological advancement has significantly impacted cardiovascular research, reshaping our understanding of immune cells' roles in the development of cardiac diseases. Therefore, it is crucial to comprehensively summarise the plasticity, heterogeneity, infiltration patterns and functional roles of immune cells using single‐cell sequencing, focusing on the dynamic regulation of immuno‐inflammatory responses across various cardiac diseases to develop more precise therapeutic targets for cardiac diseases.

## 1 Introduction

Cardiac diseases (CDs) comprise a spectrum of conditions affecting the structure and function of the myocardium, the conduction system and the heart valves, including acute myocardial infarction (AMI), heart failure (HF), arrhythmia and valvular disease [1]. Despite ongoing advancements in pharmacological treatments and device‐based therapies, CDs remain leading causes of mortality and disability worldwide, imposing a significant global health and economic burden [2]. Thus, it is crucial to identify new therapeutic targets and strategies to treat CDs. Immune cells, such as monocytes, macrophages, dendritic cells (DCs), T‐lymphocytes and regulatory T‐cells (Tregs), are dispersed throughout the healthy heart, where they exhibit high heterogeneity and interact closely with myocardial tissue [3]. These cells actively participate in both the initial inflammatory response to injury and subsequent tissue repair [4]. Studies have increasingly highlighted macrophages, the most abundant immune cells in cardiac tissues, as central regulators of immune activity. Macrophages contribute to heart development, homeostasis, and disease by sensing pathogens, clearing necrotic cells, modulating inflammation and promoting tissue repair [3, 5, 6]. Although DCs share transcriptional similarities with macrophages, they perform distinct immunological functions. DCs primarily initiate immune activation by presenting antigens to T cells, though they exhibit weaker phagocytotic [7]. Tregs, a specialised subset of T cells, play an immune suppressive role, helping to regulate the immune response and prevent excessive inflammation [8]. With the advent of single‐cell sequencing (SCS) technology, individual immune cells, including macrophages, DCs and T cells, have been analysed in a high‐resolution context, revealing significant heterogeneity and diversity in the genetic information expressed among cell subtypes [9]. The emergence of SCS technology, particularly single‐cell RNA sequencing (scRNA‐seq), has enabled high‐resolution analysis of individual immune cells, such as macrophages, DCs and T cells. This technology has revealed significant genetic heterogeneity and diversity among cell subtypes, identifying rare cell populations and providing insights into cellular diversity, intercellular communication and potential biomarkers. These discoveries have proven essential for elucidating the immune landscape of CDs and for identifying novel therapeutic targets [10, 11]. Recent advances in scRNA‐seq have provided important insights into immune cells' role in CDs such as myocardial infarction (MI), HF and arrhythmia, with growing enthusiasm for research in this field [12]. This aims to elucidate how different immune cell types and their subtypes contribute to the pathogenesis and prognosis of CDs, with a focus on leveraging scRNA‐seq to identify novel biomarkers and therapeutic targets for these conditions.

### 1.1 Development and Application of Single‐Cell Sequencing Technology

Human tissue systems are consists of various cell types, each with a unique transcriptome. Traditional bulk sequencing methods only provide an average expression profile across large cell populations, which masks intra‐cell‐type variability and introduces interpretive errors. Technological advances have now enabled researchers to uncover this hidden heterogeneity, underscoring the need for high‐resolution, single‐cell‐level analyses [13, 14, 15]. SCS involves isolating individual cells from tissues or a large cell populations and performing high‐throughput sequencing of their genomes (DNA), transcriptomes (RNA), epigenomes or spatial transcriptomes. Today, all scRNA‐seq that has been developed shares a general experimental workflow that includes cell isolation and capture, reverse transcription and cDNA amplification, library preparation, sequencing and data analysis—with single‐cell capture, reverse transcription and cDNA amplification remaining the most technically challenging steps [16, 17]. The main techniques for single‐cell isolation and capture include gradient dilution, fluorescence‐activated cell sorting (FACS), microfluidics and laser capture microdissection [18]. In recent years, the 10× genomics platform has become widely adopted due to its high‐throughput and cost‐efficiency, making it particularly suitable for large‐scale, rapid sample analysis [19]. In contrast, Smart‐seq2 excels at capturing low‐abundance transcripts, making it ideal for deep profiling of rare cell populations in complex samples [20]. Based on the 10× genomics platform, the chromium system was developed to streamline library preparation and sequencing. This system generates libraries compatible with Illumina sequencing and utilises a dedicated software suite for data processing, including barcode assignment, noise reduction and demultiplexing, offering faster and more efficient performance than droplet‐based systems [21]. By overcoming the limitations of traditional cell‐level approaches, scRNA‐seq allows researchers to uncover functional characteristics and intercellular heterogeneity across different developmental and pathological stages. Notably, scRNA‐seq enables high‐resolution transcriptomic analysis of individual cells, offering powerful tools to dissect cellular heterogeneity, uncover novel subtypes, identify new molecular markers and reconstruct developmental trajectories [22, 23].

In cardiac immunology research, numerous studies have shown that, in addition to cardiomyocytes and fibroblasts, the heart in its homeostatic state contains several subpopulations of resident immune cells, such as CC chemokine receptor 2‐negative and positive macrophages. Following MI, a significant influx of immune cells occurs. In the early phase, these infiltrating immune cells primarily clear necrotic cells through phagocytosis while secreting proteases and inflammatory cytokines such as matrix metalloproteinases (MMPs), IL‐1β and CCL2 to amplify the inflammatory response. As the infarction progresses (3–7 days post infarction), neutrophils gradually decline, and monocytes accumulate and polarise into reparative macrophages, which secrete anti‐inflammatory and pro‐repair factors, including transforming growth factor‐β (TGF‐β), IL‐10 and vascular endothelial growth factor (VEGF), to promote tissue healing. However, if inflammation persists, it may lead to irreversible myocardial damage [3]. Further investigation into immune cell subsets and their dynamic functions may reveal novel therapeutic strategies for CDs [3]. With the emergence of spatial transcriptomics, researchers have been able not only to validate the accuracy of scRNA‐seq results but also to gain additional insights into the temporal and spatial distribution of specific cell types. This integrated approach helps map the spatiotemporal dynamics of immune cells after MI, providing essential clues for the development of novel biomarkers and therapeutic interventions [24]. Similarly, Kuppe et al. [25] combined SCS with spatial transcriptomics to generate a high‐resolution map of MI regions—including ischemic, border, remote and fibrotic zones—highlighting the spatial distribution and gene expression patterns of different cell types. Their work further elucidated the spatiotemporal features of scar formation and cardiac remodelling. Thanks to scRNA‐seq's unparalleled resolution and precision in assessing cellular heterogeneity, its ability to identify new cell populations and its capacity to capture dynamic changes during cellular differentiation, this technology has transformed cardiovascular research [26]. The following sections will highlight recent progress in applying SCS to investigate the role of immune cells in the pathogenesis and resolution of CDs.

## 2 Advances in scRNA‐Seq‐Based Technology for Exploring Immune Cells in CDs

### 2.1 Myocardial Infarction

**FIGURE 1:**
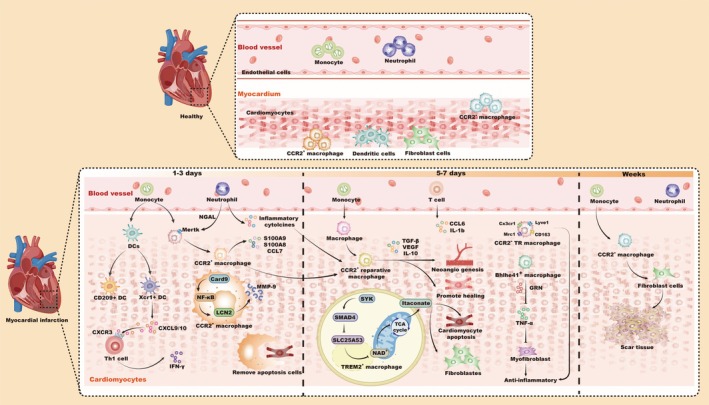
Immune cell heterogeneity in cardiac homeostasis and post‐AMI. During homeostasis, immune cells in heart tissue are primarily composed of TR CCR2^+^ macrophages, CCR2^−^ macrophages and DCs. During post‐AMI, immune cell populations, particularly macrophage subsets, undergo dynamic, time‐dependent changes, transitioning from a pro‐inflammatory to an anti‐inflammatory and reparative phenotype. CARD9, caspase‐recruitment domain family member 9; DCs, dendritic cells; LCN2, lipocalin 2; Mertk, myeloid‐epithelial‐reproductive tyrosine kinase; MI, myocardial infarction; MMPs, matrix metalloproteinases; NGAL, neutrophil gelatinase‐associated lipocalin; TGF‐â, transforming growth factor â; TR, tissue‐resident.

Immune cells residing within cardiac tissue, including monocytes and macrophages, play critical roles throughout the different stages of MI, from the inflammatory to the proliferative and maturation stages. Macrophages are the most abundant immune cell type within cardiac tissue [4]. Using the 10× Genomics platform, scRNA‐seq transcriptomic analysis of healthy mouse cardiac tissue has identified a variety of cell types, including cardiomyocytes, as well as non‐myocyte cells like fibroblasts, endothelial cells and major immune cell subsets. These cell types display complex heterogeneity, contributing to the maintenance of cardiac homeostasis and playing key roles in the pathophysiology of CDs (Figure 1) [26, 27]. Therefore, elucidating the infiltration dynamics, phenotypic transitions and functional heterogeneity under both homeostatic and ischemic conditions is essential. Single‐cell and spatial transcriptomic technologies have enabled researchers to construct high‐resolution atlases of immune cell populations at various time points after MI. These studies have unveiled the spatiotemporal dynamics of massive immune cell infiltration, including macrophages, neutrophils and DCs, in the infarcted region during the early stages of MI. They also offer comprehensive analyses of necrotic cell clearance, immune recruitment, myofibroblast activation and neovascularization during cardiac remodelling, thereby providing mechanistic insights into post‐MI repair (Figure 1) [25]. A recent study performed scRNA‐seq analysis on myocardial tissue cells in mice after MI. Twelve gene clusters corresponding to major immune cell populations and their subpopulations were identified, among which macrophages were the most abundant cells, reaching a peak at 3 days after MI, and were divided into six clusters according to different surface markers to perform antigen presentation, leukocyte chemotaxis, anti‐inflammatory and pro‐inflammatory functions, respectively. As MI progressed from Day 7 to 14, the number of inflammatory cells decreased, with macrophages shifting from pro‐inflammatory to anti‐inflammatory phenotypes, aiding tissue repair. A macrophage subpopulation promoting fibrosis during cardiac remodelling was also detected, indicating its role in cardiac remodelling [28]. From Day 7 to 14, the number of inflammatory cells declined, and macrophages progressively transitioned from pro‐inflammatory to anti‐inflammatory phenotypes, contributing to tissue repair. A fibrosis‐associated macrophage subpopulation was also detected, implicating its role in adverse remodelling. These results align with previous studies showing that the early phase of MI involves intense immune infiltration and inflammatory cytokine secretion. Timely resolution of inflammation is vital for optimal healing. Jung et al. [24] employed SCS alongside spatial transcriptomics to map the spatial positioning of various cell types at different stages post‐MI, identifying a reparative TREM2^+^ macrophage subpopulation that became dominant during the late phase. Further mechanistic studies demonstrated that TREM2^+^ macrophages regulate immunometabolism via efferocytosis‐linked signalling. Specifically, TREM2^+^ macrophages suppressed SLC25A53 transcription through the SYK–SMAD4 axis following efferocytosis, thereby limiting mitochondrial NAD transport and tricarboxylic acid (TCA) cycle flux. This shift enhanced production of the anti‐inflammatory metabolite itaconate. In vitro, itaconate inhibited cardiomyocyte apoptosis and promoted fibroblast proliferation. TREM2 overexpression in macrophages significantly improved cardiac repair and restored left ventricular function (Figure 1) [29]. The phenotypic transition of macrophages from a pro‐inflammatory to an anti‐inflammatory state is critical for MI healing. Myeloid‐epithelial‐reproductive tyrosine kinase (Mertk), a marker of M2 macrophages, is essential for clearing apoptotic cardiomyocytes. A Mertk deficiency leads to delayed inflammation resolution, worsened remodelling and impaired cardiac function [30]. Interestingly, neutrophils in the early post‐MI phase modulate macrophage polarisation toward an M2 phenotype by secreting neutrophil gelatinase‐associated lipocalin (NGAL), which upregulates macrophage Mertk expression. M2 macrophages subsequently release anti‐inflammatory cytokines (e.g., TGF‐β and IL‐10) and VEGF to promote angiogenesis and tissue healing [3, 31]. Distinct macrophage ontogenies also exhibit unique transcriptional profiles. Using scRNA‐seq and gene enrichment analysis, researchers differentiated tissue‐resident (TR) macrophages (low CCR2 expression) from monocyte‐derived CCR2^+^ macrophages. TR macrophages downregulate inflammatory genes (CCR2 and Ly6C2) and upregulate reparative genes (LYVE1, CX3CR1, CD163 and Mrc1). Gene set enrichment analysis (GSEA) showed that TR macrophages are involved in cell proliferation and growth factor signalling (e.g., TGF‐β), whereas CCR2^+^ macrophages activate pro‐inflammatory pathways [32]. These findings suggest that TR macrophages self‐renew and serve as anti‐inflammatory regulators during cardiac repair.

Emerging studies are using scRNA‐seq to uncover therapeutic targets. One study identified a monocyte‐derived Bhlhe41^+^ macrophage subpopulation (Mϕ3), which peaks at Day 7 post‐MI and disappears by Day 14. These macrophages, enriched in infarct‐progression zones, were associated with sodium‐glucose cotransporter 2 (SGLT2) inhibitor treatment and engaged in lipid and collagen metabolism. They improved cardiomyocyte survival by regulating GRN and TNFR1 signalling, while suppressing myofibroblast overactivation (Figure 1) [33]. In contrast, some immune responses are maladaptive. Caspase‐recruitment domain family member 9 (CARD9) is a pro‐inflammatory mediator enriched in F4/80^+^ macrophages. CARD9 knockout mice exhibited improved cardiac function, reduced cardiomyocyte apoptosis and lower MMP2/3/9 levels. Molecular and scRNA‐seq studies revealed that CARD9 deficiency inhibited NF‐κB activation and downstream LCN2 expression, attenuating inflammation and fibrosis (Figure 1) [28, 34]. While the role of DCs in MI remains incompletely understood, recent scRNA‐seq profiling has defined six DC subclusters, including CD209^+^, Xcr1^+^ and migratory (Fscn1^+^) subsets. Clusters 2–4 predominated in homeostasis (93.8% of DCs) but declined by Day 5 post‐MI, while Fscn1^+^ DCs and macrophages (Ms4a7^+^ and Fcrls^+^) increased later [24]. This suggests that migratory Fscn1^+^ DCs may suppress immune responses during late remodelling. Moreover, XCR^+^1cDC1 cells express the chemokines CXCL9 and CXCL10, which activate Th1 cells—the primary CD4 T‐cell subset recruited to the ischemic heart via CXCR3 chemokine signalling. This promotes the release of large amounts of IFN‐γ, which drives unfavourable cardiac remodelling after ischemic myocardial injury (Figure 1) [35]. It is crucial to recognise the significant role that T cells play important role in both initiating and regulating the immune response, as well as their involvement in cardiac remodelling following MI [36]. To further investigate T cell heterogeneity post‐MI, scRNA‐seq analysis at Days 3 and 7 post‐MI revealed robust T cell extravasation and identified four subsets: primary, effector, regulatory (Tregs) and NK cells. Effector T cells upregulated pro‐inflammatory mediators, including CCL6 and IL‐1β [32]. Tregs, by contrast, secrete IL‐10 and TGF‐β, maintaining immune homeostasis and cardiac function [32]. These results highlight T cells as potential targets for immunomodulation post‐MI [37].

### 2.2 Heart Failure

Following myocardial injury, the persistence or dysregulation of inflammatory cells can drive chronic, pathological inflammation and adverse ventricular remodelling, leading to changes in ventricular size and function and ultimately progressing to HF [38, 39]. ScRNA‐seq can detect gene expression changes within immune cells—including macrophages, lymphocytes and DCs—in the heart and vasculature, revealing how these immune populations regulate and influence HF progression in the context of cardiac disease [40].

**FIGURE 2:**
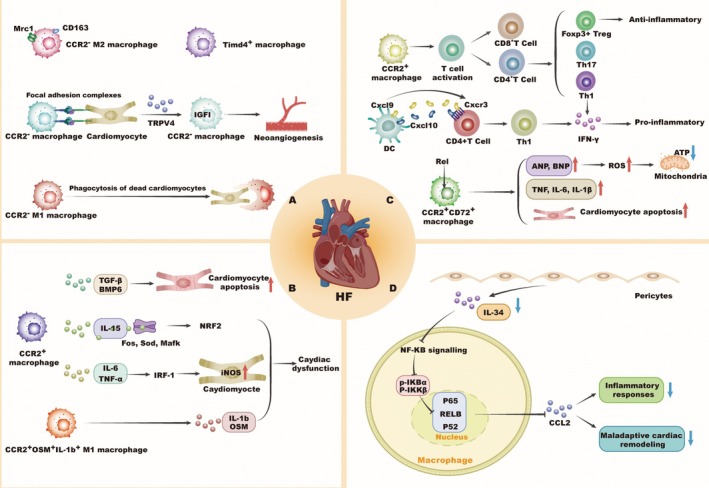
Insights into the roles of distinct immune cell subsets in HF through SCS. (A) Anti‐inflammatory macrophages, identified by specific molecular markers, play a protective role in HF by promoting tissue repair and resolving inflammation, thereby slowing disease progression. (B) CCR2^+^ macrophage subsets worsen HF by promoting cardiomyocyte apoptosis, producing iNOS, and releasing inflammatory cytokines. (C) DCs, different types of T cells, and CCR2^+^CD72^+^ macrophages play a role in the progression of HF through their pro‐inflammatory activities and ability to present antigens. (D) After myocardial ischaemia–reperfusion, pericytes release IL‐34, which activates the inflammatory NF‐κB signalling pathway. This leads to an increase in the expression of inflammatory cytokines and contributes to adverse cardiac remodelling. HF, heart failure; IL‐34, interleukin‐34; IRF1, interferon regulatory factor 1; Osm, oncostatin M; TRPV4, transient receptor potential vanilloid 4.

Extensive research has demonstrated the activation of multiple immune cells in the hearts of diseased mice. By utilising scRNA‐seq, researchers have accurately identified immune cell subpopulations in remodelling hearts, which is instrumental for analysing the dynamic changes in inflammatory and immune responses at different stages in pressure overload (TAC) mouse hearts. This approach has deepened our understanding of the complexity of immune regulation in failing heartsHF [41]. For instance, scRNA‐seq analysis of monocyte/macrophage clusters in TAC‐induced HF hearts revealed multiple subsets, including CCR2^−^ M2‐like reparative macrophages, expressing M2‐like markers CD163 and Mrc1, as well as major histocompatibility complex (MHC) Class II molecules. Additionally, CCR2^−^ M1‐like phagocytic monocytes/macrophages participate in cardiac homeostasis by engulfing dead cardiomyocytes (Figure 2A). A pro‐inflammatory M1‐like subset—CCR2^+^Osm^+^IL‐1β^+^ monocyte/macrophages—was also identified, marked by high expression of IL‐1β and oncostatin M (Osm) (Figure 2B) [41]. In ischemic HF induced by MI, a combination of cell tracing and scRNA‐seq highlighted macrophage diversity and functional heterogeneity. The damaged heart contained Timd4 resident macrophages and CCR2 recruited cells. A marked reduction in resident macrophages within the infarcted area suggested their replacement by recruited monocyte‐derived macrophages [42]. Similarly, in a hyperlipidemia‐induced model of HF with preserved ejection fraction (HFpEF), scRNA‐seq revealed that Timd4 macrophage subpopulations highly express anti‐inflammatory molecules (Figure 2A), while CCR2 macrophages upregulated pro‐inflammatory genes, such as TGF‐β and BMP6, Fos, Myc, Irf1, and TNF‐α, IL‐15, and IL‐6. TGF‐β‐BMP6 signalling was shown to promote apoptosis and fibrosis, contributing to extracellular matrix remodelling, myocardial hypertrophy and HF. The interaction between IL‐15 and oxidative stress response genes such as Fos, Sod and Mafk may activate the NRF2‐oxidative response pathway, thereby contributing to cardiac dysfunction. Exposure to TNF‐α, IL‐6, IL‐15 or their combination significantly upregulated Irf1, Angptl, Fos and Cpt1b expression. At the protein level, the protein level, these cytokines induced IRF1, which in turn activated iNOS in cardiomyocytes, leading to cardiac dysfunction (Figure 2B). Inhibiting inflammatory cytokine production and preserving Timd4 macrophage homeostasis may mitigate cardiac dysfunction [43]. Studies have shown that CCR2^−^ TR macrophages play a key regulatory role in cardiac remodelling during chronic HF. In a study using a dilated cardiomyopathy mouse model combined with a macrophage depletion model, it was found that CCR2^−^ macrophages form stable interactions with adjacent cardiomyocytes through focal adhesion complexes. These macrophages sense mechanical stretch and enhance the expression of transient receptor potential vanilloid 4 (TRPV4) mRNA. Treatment with a TRPV4 inhibitor demonstrated that TRPV4 activates CCR2^−^ macrophages by regulating insulin‐like growth factor 1 (IGF1) expression, which in turn promotes coronary angiogenesis and supports adaptive remodelling and survival in chronic failing hearts (Figure 2A) [44]. Notably, SCS of HF models uncovered macrophage heterogeneity including a CCR2^+^ sunset highly expressing CD72, a marker of inflammatory macrophages. The expression of CD72 in macrophages is regulated by the Rel transcription factor. When cardiomyocytes were co‐cultured with bone marrow‐derived macrophages (BMDMs) overexpressing Rel, there was an increase in the expression of cardiac stress markers (ANP and BNP) and oxidative enzymes (such as Cybb, Sod2, Gsr and Prdx5) in the cardiomyocytes. This also led to higher cytosolic ROS levels, increased mitochondrial content, but reduced ATP production. Simultaneously, several inflammatory pathways, including TNF and IL‐6, were activated in the macrophage‐cardiomyocyte interactions, resulting in the release of large amounts of inflammatory cytokines (TNF, IL‐6 and IL‐1β), which promoted cardiomyocyte apoptosis. Furthermore, the detrimental effects of Rel were found to be dependent on CD72 expression, indicating that CD72 macrophages may be a potential therapeutic target for cardiac injury (Figure 2C) [45]. To further investigate immunoregulatory strategies for preventing and reversing adverse cardiac remodelling and HF in post‐MI patients after clinical treatment, researchers used scRNA‐seq and identified IL‐34 as an inflammation‐regulating factor produced by pericytes, upregulated after myocardial ischemia/reperfusion (IR) injury. IL‐34 gene knockout significantly reduced post‐IR fibrosis, dysfunction and inflammation. Mechanistically, IL‐34 deletion suppressed both classical and non‐classical NF‐κB signalling, reducing phosphorylation of IKKβ and IκBα, and downregulating NF‐κB p65, RelB and p52, thereby lowering CCL2 expression and macrophage recruitment (Figure 2D) [46]. ScRNA‐seq further revealed that during the early stages of TAC, CCR2 monocyte‐derived macrophages infiltrate the damaged heart and trigger T cell activation, replacing TR macrophages (Figure 2C). Blocking CCR2 macrophage recruitment reduced T‐cell expansion, attenuated cardiac remodelling and improved cardiac function, highlighting CCR2 macrophages as a key immunotherapeutic target for treating pressure overload‐induced HF. Similarly, inhibiting M1 macrophage polarisation has shown cardioprotective effects after MI [4]. Single‐cell studies have identified new immunotherapy targets. For example, miR‐21 knockout in pressure overload models suppressed TR macrophages polarisation toward the M1 phenotype and promoted M2‐like activation. Moreover, reduced miR‐21 impeded the fibroblast‐to‐myofibroblast transition, mitigating fibrosis. These findings emphasise the role of TR macrophages in pressure overload‐induced dysfunction and suggest miR‐21 as a key modulator [47]. T‐cell expansion is a critical step in the transition from TAC to HF [48]. ScRNA‐seq has identified both CD8 (Clusters 6 and 15) and CD4 (Clusters 9 and 13) T cells in the heart during HF, with CD4 T cells playing a more central role in disease progression. Upon antigen activation, T cells in the damaged myocardium expand and upregulate activation markers like CD69 and Ccr7. Notably, CD8 T cells (Cluster 15) are associated with lymphocyte activation, differentiation and proliferation [41]. Among these, CD4 T cell subsets, including T helper cells (Th1 and Th17) and Tregs, have dual effects on cardiac remodelling (Figure 2C) [49, 50]. Tregs are a distinct cluster of T cells, with Cluster 14 expressing key Tregs markers such as Foxp3, Tnfrsf18 (GITR) and Ctla4 mRNA. They also uniquely express the programmed cell death receptor 1 (PD‐1), contributing to their anti‐inflammatory role (Figure 2C) [41, 51]. In response to pressure overload, key molecules specific to certain immune cell subsets are upregulated, such as Oncostatin M in pro‐inflammatory macrophages and PD‐1 in Tregs. The interaction between PD‐1 and its ligand inhibits T cell activity, helping to maintain immune balance. However, in clinical practice, HF patients undergoing anti‐PD‐1 cancer immunotherapy may experience T‐cell reactivation and proliferation, worsening cardiac toxicity [41, 52, 53]. Recent research has explored the roles of T cells and DCs in ischemic HF immunotherapy. Th1 cells, in particular, are crucial for recruiting monocyte/macrophage during the acute phase post‐MI by producing the pro‐inflammatory cytokine IFN‐γ (Figure 2C) [50]. In TAC‐induced HF, CXCR3 Th1 cell recruitment is linked to adverse remodelling [54]. In a HFD‐CHD mouse model, CXCL9 and CXCL10 promote CD4 Th1 cell recruitment to the ischemic myocardium, modulating immune responses. In parallel, XCR1^+^ cDC1 activation contributed to adverse remodelling post‐injury (Figure 2C) [35].

### 2.3 Arrhythmia

Disruptions in the rhythmic excitability of cardiomyocytes or conduction system dysfunction can lead to arrhythmias, which are the leading cause of sudden cardiac death, with atrial fibrillation (AF) being the most common sustained arrhythmia [55]. Increasing evidence shows that during AF, significant changes occur in the immune system, including alterations in immune cell composition, quantity and immune molecule levels. These immune changes interact with atrial structural and electrical components, a phenomenon referred to as immune remodelling. Understanding the immune system's critical role in AF pathophysiology sheds light on the complex interplay between atrial electrophysiology, electrical remodelling, neural remodelling and immune remodelling [56].

**FIGURE 3:**
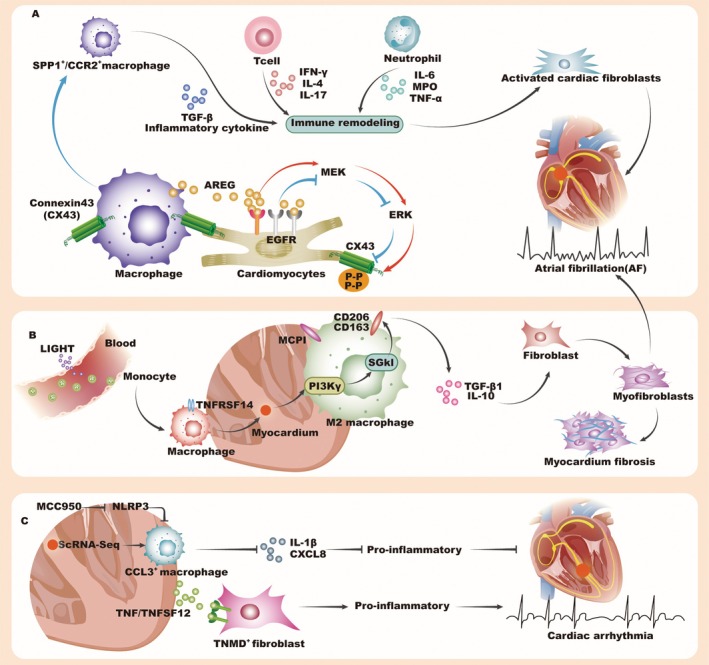
Immune cell subsets in arrhythmias. Their role in regulating cardiac fibrosis and electrical conduction. (A) In a healthy heart, cardiac macrophages support normal cardiac impulse conduction by producing AREG, which promotes CX43 phosphorylation and helps establish stable gap junctions with CX43. However, when the AREG gene is deleted in cardiac macrophages, these gap junctions become disorganised, resulting in arrhythmias. This deletion also triggers a significant increase in SPP1^+^/CCR2^+^ macrophages, while other immune cells, such as T cells and neutrophils, undergo immune remodelling, further exacerbating myocardial fibrosis and increasing susceptibility to arrhythmias. (B) Macrophage migration and cardiac fibrosis: Macrophages infiltrate the ventricles and atria, driving the transformation of fibroblasts into myofibroblasts and increasing collagen synthesis. This process results in cardiac fibrosis and heightened susceptibility to atrial fibrillation. (C) ARVC and pro‐inflammatory macrophages: In ARVC, the right ventricle is populated with CCL3^+^ pro‐inflammatory macrophages and tenomodulin (TNMD) fibroblasts. Their interaction intensifies the inflammatory response, leading to the development of malignant arrhythmias. AF, atrial fibrillation; AREG, amphiregulin; ARVC, arrhythmogenic right ventricular cardiomyopathy; CX43, connexin 43; NLRP3, NOD‐like receptor protein 3.

Studies have shown that alterations in the expression of ion channel and gap junction‐related genes, such as Connexin 43 (CX43), are associated with arrhythmogenicity in HF patients. CX43, a key component of gap junctions, regulates intercellular electrical conduction in the myocardium [57, 58]. Utilising SCS and single‐nucleus transcriptomic analysis of six regions of the adult heart, researchers identified cellular heterogeneity in cardiomyocytes, pericytes and fibroblasts, and cardiac resident macrophages with both inflammatory and protective transcriptional profiles [59]. To assess the role of immune cells in arrhythmias caused by acute stress in the right ventricle (RV), researchers performed pulmonary artery banding in mice to induce RV pressure overload. The stressed RV shows an accumulation of immune cells, including macrophages, CD4 and CD8 T cells, and B cells. The findings show that cardiac macrophages enhance communication between cardiomyocytes through gap junctions, maintaining proper cardiac impulse conduction. Depletion of these macrophages led to severe arrhythmias and sudden death [60]. A key macrophage‐derived mediator, amphiregulin (AREG), promotes CX43 phosphorylation via the EGFR/MEK/ERK pathway, thereby stabilising gap junction formation and supporting electrical coupling. In the absence of AREG, gap junction disorganisation leads to fatal arrhythmias under stress conditions such as RV pressure overload or β‐adrenergic stimulation, including ventricular arrhythmias and sinus arrest. These findings suggest that AREG in cardiac macrophages acts as a critical regulator of cardiac impulse propagation and may serve as a potential therapeutic target for arrhythmia prevention (Figure 3A) [60].

Hulsmans et al. [61] conducted scRNA‐seq on atrial tissue from patients with persistent AF and control individuals, identifying six major non‐cardiomyocyte clusters: lymphocytes, mononuclear phagocytes and DCs (MP/DC), endothelial cells, fibroblasts, mural cells and neutrophils. In AF patients, there was a notable increase in CCR2^+^ inflammatory monocytes and SPP1^+^ macrophages, suggesting that AF is associated with an increase in macrophages rather than DCs or other cell types (Figure 3A). Additionally, SPP1 interacts with integrins such as α4β1, αvβ3 and α9β1, which are broadly expressed in cardiac immune and stromal cells. This interaction triggered the release of inflammatory cytokines and extracellular matrix proteins from atrial cells. Through the TGF‐β pathway, SPP1^+^ macrophages drive fibroblast activation and tissue remodelling, ultimately promoting AF by altering atrial electrophysiology (Figure 3A). These findings indicate that macrophages expressing SPP1 exacerbate atrial inflammation and fibrosis by creating an electrophysiological substrate conducive to AF development. Unexpectedly, analysis of peripheral blood mononuclear cells (PBMCs) from AF patients showed increased mRNA levels of LIGHT (TNFSF14) and its receptor TNFRSF14. LIGHT promotes cardiac fibrosis and AF susceptibility. Flow cytometry and SCS revealed LIGHT expression in lymphocytes and NK cells, with TNFRSF14 in macrophages. Tail vein injection of recombinant LIGHT induced macrophage infiltration, fibrosis and upregulated MCP1 and M2 markers in cardiac tissue. Transcriptomic analysis identified the PI3Kγ/SGK1 pathway, confirming LIGHT activates this pathway, promoting M2 macrophage polarisation and fibroblast transformation, leading to cardiac fibrosis and increased AF risk (Figure 3B) [62].

Inflammation is also implicated in the progression of arrhythmogenic right ventricular cardiomyopathy (ARVC), although the precise regulatory mechanisms remain unclear. SCS in ARVC patients revealed an enrichment of CCL3 pro‐inflammatory macrophages and TNMD‐expressing fibroblasts in the right ventricle. A strong interaction between these macrophages and fibroblasts is mediated by TNF/TNFSF12 signalling, amplifying inflammatory responses via the MK pathway. NLRP3, identified as the transcription factor for CCL3 macrophages, correlates with increased IL‐1β and CXCL8 expression, driving inflammation. Inhibition of NLRP3 with MCC950 in Dsg2mut/mut ARVC mice significantly reduced right ventricular dilation, dysfunction and malignant arrhythmias, highlighting NLRP3 as a potential therapeutic target for mitigating cardiac inflammation and remodelling in ARVC (Figure 3C) [63].

### 2.4 Valvular Heart Disease

**FIGURE 4:**
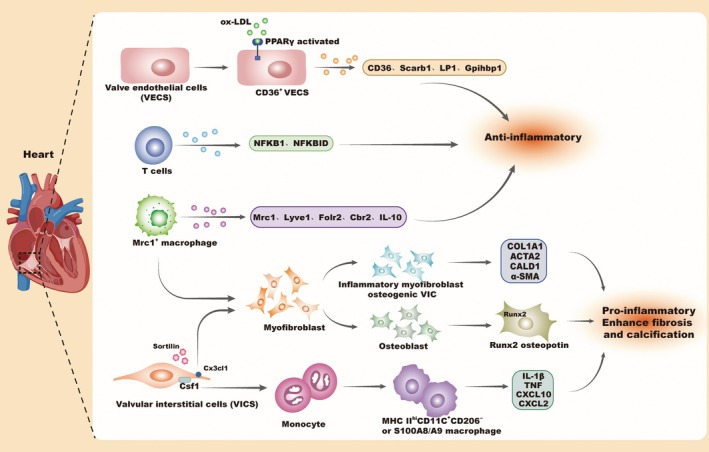
Immune cell infiltration and cellular transformation in cardiac valvular disease. VECs, valve endothelial cells, VHD, valvular heart disease, VICS, valvular interstitial cells, RUNX2, runt‐related transcription factor 2.

Valvular heart disease (VHD) is a prevalent cardiovascular disorder involving structural or functional abnormalities of the heart valves. The mitral valve (MV) is most frequently affected, followed by the aortic valve (AOV) [64]. The phenotypes and transcriptional profiles of healthy, mature heart valve cells are diverse, consisting mainly of valvular interstitial cells (VICs), valvular endothelial cells (VECs) and immune cells, such as macrophages, T cells and DCs [65]. Macrophages, which originate from the endocardium, play a key role in valve remodelling during development [66]. VICs constitute a heterogeneous population of fibroblasts, including a small subset of myofibroblasts and SMCs, which may act as the primary source of myofibroblasts and osteoblasts, driving valvular calcification in pathological conditions. VECs regulate leaflet stability and inflammation, contributing to both valvular homeostasis and disease. They also influence VIC function through paracrine signalling [67]. The aetiology and pathogenesis of calcific aortic valve disease (CAVD) are complex, with inflammation playing a central role in disease progression [68]. CAVD encompasses multiple pathological processes, including endothelial dysfunction, immune cell infiltration, valvular fibrosis, calcification and extracellular matrix remodelling. These processes activate VICs into myofibroblasts, which subsequently differentiate into osteoblast‐like cells characterised by elevated expression of Runt‐related transcription factor 2 (RUNX2) and osteopontin. This cascade promotes progressive fibrosis, calcification, valve thickening and ultimately aortic valve stenosis (AVS) (Figure 4) [68, 69]. ScRNA‐seq has characterised the immune landscape of calcified aortic valves, identifying macrophages as the dominant immune cell type. S100A8 and S100A9, highly expressed in macrophages and monocytes, were associated with increased M1 macrophages, Tregs, plasma cells and NK cells. S100A8 and S100A9, highly expressed in macrophages and monocytes, are associated with increased infiltration of M1 macrophages, Tregs, plasma cells and NK cells [70]. In a hyperlipidemia‐induced aortic valve calcification model, scRNA‐seq revealed two distinct macrophage populations: MHCIICD11c and MHCIIMrc1 (CD206^+^). Pro‐inflammatory MHCIIhiCD11cCD206^−^ macrophages accumulated in LDL‐rich areas, expressing high levels of IL‐1b, TNF, CXCL10, CXCL2 and Itgax, while anti‐inflammatory MHCIIMrc1 macrophages expressed IL‐10 and other protective genes. VICs expressed Csf1 and Cx3cl1, contributing to monocyte recruitment and inflammation. Additionally, a Cd36‐expressing subpopulation of VECs showed enrichment in PPARγ pathway genes. LDL accumulation and its conversion to ox‐LDL triggered PPARγ activation, which upregulated lipid metabolism genes in VECs, protecting valves from excessive inflammation and calcification in early disease stages. PPARγ knockout in human aortic VECs increased expression of pro‐inflammatory genes, highlighting its anti‐inflammatory role (Figure 4) [71]. Clinical data suggest pioglitazone, a PPARγ agonist, used to treat diabetes, effectively reduces aortic valve inflammation. This points to the potential therapeutic benefit of combining lipid‐lowering drugs with pioglitazone to manage early aortic valve inflammation. Another study used scRNA‐seq to analyse human VICs cultured under osteogenic conditions, identifying 12 distinct phenotypes and their heterogeneity, including a newly discovered inflammatory myofibroblast‐osteogenic VIC (IMO‐VIC) phenotype. Activated by sortilin, these IMO‐VICs secrete COL1A1, ACTA2, CALD1 and α‐SMA, playing a key role in CAVD progression. Inhibiting sortilin specifically prevents the transformation of VICs into myofibroblasts, potentially slowing or halting CAVD progression (Figure 4) [72].

Recent studies have increasingly focused on bicuspid aortic valve (BAV), a major risk factor for calcific CAVD [73]. To explore immune cell infiltration in BAV, Lyu et al. [74] performed scRNA‐seq on BAV specimens from patients with aortic stenosis, revealing a significant increase in lymphocytes during severe calcification. The analysis uncovered functional differences among subpopulations, including a rare subset of lymphocytes with high expression of inhibitory molecules such as NFKB1 and NFKBID, which regulates NF‐κB activity [75, 76]. These cells, termed inhibitory T cells, exhibit immunosuppressive properties. Researchers suggest that this subset may represent the body's attempt to maintain homeostasis in response to heightened inflammation in severely calcified BAV (Figure 4). The study also highlighted that MRC1 macrophages (M2 type) in calcified BAVs can shift towards a pro‐inflammatory phenotype and differentiate into myofibroblasts and osteoblasts. Proteomic analysis further confirmed that the phenotype of human VICs can transition into myofibroblasts, mediated by sortilin, contributing to valvular fibrosis and calcification (Figure 4).

## 3 The Role and Potential of Immunometabolic Interactions in Cardiac Disease

Growing evidence suggests that the crosstalk between inflammation and metabolism constitutes a central mechanism in the pathogenesis of cardiovascular disease, with immune cells—particularly macrophages—acting as key modulators in this dynamic interplay. Zhang et al. [77] demonstrated that macrophages are not only critical responders to cardiac injury but also contribute to cardiac homeostasis and myocardial repair. Their functional states are closely shaped by the local metabolic microenvironment [77]. Recent advances in spatial single‐cell transcriptomics have revealed pronounced metabolic remodelling in surviving cardiomyocytes following MI, including increased glycolysis and reduced fatty acid oxidation. These metabolic adaptations help sustain cellular energy under hypoxic conditions and are tightly linked to immune cell activation [78]. Notably, a specific subset of TREM2^+^ macrophages has been shown to mediate cardiac repair by regulating mitochondrial NAD^+^ transport through the solute carrier SLC25A53. This disrupts the TCA cycle, promotes itaconate production and induces immunometabolic reprogramming, highlighting TREM2^+^ macrophages as promising dual targets for metabolic and immune modulation [29]. Under metabolic stress, excess lipid accumulation within cardiomyocytes can activate oxidative and endoplasmic reticulum stress pathways, which in turn dysregulate lipid metabolism in macrophages and trigger inflammatory cascades involving NF‐κB and JNK pathways. These responses exacerbate cardiac dysfunction. Importantly, SGLT2 inhibitors have been shown to alleviate local inflammation and insulin resistance by promoting fatty acid utilisation and inducing M2‐like macrophage polarisation, thereby offering a novel therapeutic avenue for immunometabolic regulation [43]. Taken together, the metabolic state of immune cells is tightly coupled with their inflammatory profiles, while immunometabolic reprogramming profoundly influences cardiomyocyte survival and function. This reciprocal interaction between metabolism and immunity is emerging as a key breakthrough point in cardiovascular research and therapy [79]. However, resolving cell‐type‐specific metabolic heterogeneity and translating metabolic targets into effective interventions remain major challenges. Addressing these questions will require continued integration of spatial multi‐omics and human‐relevant model systems such as cardiac organoids.

## 4 Translational Bottlenecks and Strategic Solutions for Single‐Cell Technologies in Cardiac Disease

ScRNA‐seq enables the identification of key cellular populations involved in the development and progression of cardiac diseases by revealing their spatial distribution, lineage trajectories, immune activity and functional characteristics [80]. For example, in a murine MI model, studies integrating spatial transcriptomics with single‐cell approaches have uncovered significant dynamic remodelling of the macrophage lineage after MI. Notably, Trem2^^hi^ macrophages emerged as a dominant subpopulation during the later stages. Intriguingly, administration of soluble TREM2 protein in vivo significantly improved cardiac structure and function, suggesting this macrophage subset may serve as a promising target for post‐MI immunomodulatory therapy [24]. Another study employing spatial transcriptomics and trajectory inference identified a transient wave of Bhlhe41^+^ resident macrophages that peaked at Day 7 post‐MI. These macrophages played a key role in suppressing cardiac fibroblast activation and limiting scar formation, highlighting the spatiotemporal plasticity and functional specialisation of macrophages in MI [33]. In the context of arrhythmias, particularly AF, a snRNA‐seq study comparing left atrial tissue from AF patients and controls revealed significant transcriptomic alterations in both cardiomyocytes and macrophages. Among these, ATRNL1 was markedly upregulated in cardiomyocytes during AF and localised to intercalated discs. Functional analyses suggested its involvement in stress responses and action potential regulation, indicating a potential pathogenic role and therapeutic relevance in AF [81]. Based on these findings, this review systematically summarises macrophage‐targeted therapeutic strategies identified through SCS in various cardiac diseases, with particular emphasis on frequently reported or functionally validated macrophage subpopulations (Table 1).

**TABLE 1:** ScRNA‐seq enables discovery of macrophage‐targeted therapeutic targets in CDs.

Diseases	Cell subsets (scRNA‐seq)	Research methods/targeted strategy	Mechanism of action	Application/development phase	References
AMI	TREM2^+^ macrophages	Intravenous injection of soluble TREM2 in animals；Mac‐TREM2KO mice	Elevating itaconate levels establishes an anti‐inflammatory environment and promotes cardiac repair.	Preclinical studies, but TREM2 agonists are being tested in Phase III clinical trials for Alzheimer's disease.	[24, 29]
	CCR2^+^CARD9^+^macrophages	CARD9 knockout mice	CARD9 deficiency can inhibit NF‐κB and reduce the expression of MMP9 and LCN2.	Basic research stage	[34]
	Bhlhe41^+^ macrophages	Increase the expression of Bhlhe41	Secreting granulin (GRN) to inhibit fibroblast activation	Preclinical research phase	[33]
	CCR2^+^ macrophages	CCR2 antagonism, anti‐CCL2 antibody	Inhibiting the recruitment of inflammatory monocytes and reducing the expression of inflammation‐related genes including IL1β, CCL7/MCP3	Animal experiments, partially entering early clinical stages	[82]
	CD86^+^ macrophages	Nanotherapeutic delivery system, codeliver siSTAT1 and the small‐molecule nitro‐oleic acid (OA‐NO_2_)	Reduce the number of CD86^+^ macrophages, increase the number of Cx3cr1^+^ macrophages and recruit Tregs	Animal experiments, preclinical studies	[83]
	S100a9^+^Ly6c^+^ macrophages	CXCR2 inhibitor (SB225002)	Inflammatory factors decreased, while the proportion of the reparative Arg1^+^ macrophage increased.	Basic research validation phase	[84]
HF	Timd4^+^ macrophages	Single‐cell tracking, Cx3cr1 transgenic mouse model	Maintenance of resident macrophage functions	Basic research phase	[42]
	CCR2^+^ macrophages	Dapagliflozin.CCR2 antagonist. SGLT2‐knock out mice	Dapagliflozin Inhibiting pro‐inflammatory macrophages	Clinical application phase CCR2 antagonist development in progress	[85]
	Timd4^+^ Lyve1^+^ macrophages	Dapagliflozin	Restoring the abundance of resident macrophages and attenuating metabolic stress‐related myocardial inflammation	Preclinical mechanism research	[43]
	CCR2^−^ reparative macrophages	TRPV4 inhibitor	Regulate IGF1 expression, activate CCR2^−^ macrophages and improve cardiac function	Validated in animal models, basic research stage	[44]

	CCR2^+^CD72^+^ pro‐inflammatory macrophages	Knockout of transcription factor Rel reduces CD72 expression	Inflammatory factors TNF, IL‐6 and IL‐1β decreased, and cardiomyocyte apoptosis was alleviated.	Preclinical research	[45]
Ccrl2^+^ macrophages	α7nAChR‐specific agonist (PNU‐282987) mimics optogenetic vagus nerve stimulation (Opto‐VNS)	Reducing the accumulation of inflammatory Ccrl2^+^ macrophages through the α7nAChR‐NRF2 signalling axis alleviates cardiac hypertrophy and fibrosis	Proven to be relevant in human HF patients	[86]
Arrhythmia	CCR2^+^ SPP1^+^ inflammatory macrophages	Targeted inhibition of CCR2 or SPP1	Reducing the recruitment of inflammatory macrophages, blocking the activation of inflammatory fibroblasts	Validation was conducted in human samples, preclinical validation period	[61]
	CCL3^+^ macrophages	NLRP3 inhibitor (MCC950)	Reducing the release of inflammatory factors IL‐1β and CXCL8, alleviating cardiac inflammation and remodelling in ARVC	Preclinical research, with some already entering the preliminary clinical trial evaluation stage	[63]
	CCR2^+^ macrophages	NF‐κB inhibitor (Bay 11–7082). CCR2‐targeted deletion	Inhibiting the NF‐κB signalling pathway thereby blocks the activation of CCR2^+^ macrophages, limiting inflammatory responses, fibrofatty replacement and the occurrence of arrhythmias.	Preclinical research phase requires further safety and efficacy evaluations before advancing to the clinical stage.	[87]
	IL1B^+^ macrophages	Targeting the epidermal growth factor (EGF) signalling pathway. IL‐1 receptor inhibitors (canakinumab)	Interrupt the abnormal signal transduction between monocytes/macrophages and fibroblasts, alleviating inflammation and fibrosis	Preclinical research	[88]
Valvular heart disease	S100A8^+^/S100A9^+^ macrophages	Targeting S100A8/S100A9	Inhibiting NF‐κB activation and reducing inflammatory factor secretion	Validation in CAVD human samples	[70]
	MHCIIhiCD11cCD206^−^ macrophages	PPARγ agonist (pioglitazone)	Inhibiting the activation of NF‐κB, inducing the expression of genes related to lipid metabolism, such as Cd36.	Preclinical research	[71]
	CD301b/MGL2^+^ mononuclear macrophages	Genetically modified mice lacking CX3CR1 or CD301b/MGL2 expression. Drug‐induced cell depletion.	Pro‐inflammatory cytokines TNF and IL‐6 decreased.	Preclinical studies require further clinical trials for verification.	[89]
	MRC1^+^ (CD206) macrophages	Targeted inhibition of regulatory gene FOXC1 and the PI3K‐AKT signalling pathway	Blocking macrophage‐mesenchymal transition	Early‐stage preclinical research	[74]

Despite the remarkable progress achieved in basic research, the clinical translation of these technologies still faces numerous technical and systemic challenges. To address these issues, researchers are actively exploring potential solutions, which primarily focus on the following aspects. (1) Species differences limit translational applicability. Most current single‐cell studies rely on murine models; however, substantial interspecies differences in cardiac structure (e.g., atrioventricular proportions and conduction system), immune system (e.g., chemokine expression) and gene regulation hinder direct extrapolation to humans. For instance, murine immune cells express much higher levels of genes such as *Tlr7* and *Ifit1b*—key mediators of viral sensing and interferon responses—compared to humans, whereas human cardiomyocytes exhibit increased sensitivity to certain stress‐related genes [90, 91]. To this end, researchers have established human cardiac organoids (cardioids) integrating cardiomyocytes, cardiovascular progenitors and immune components to simulate key processes such as cardiac development, myocardial inflammation and fibrotic tissue remodelling [92]. Furthermore, organ‐on‐chip systems combining fluid dynamics with immune co‐culture provide highly biomimetic platforms for drug screening [93]. (2) Limited accessibility of high‐quality human tissue samples. Cardiac tissue samples primarily rely on transplants, autopsies or surgical discards, but ethical constraints, delayed disease staging and limited sample sources hinder the acquisition of high‐quality specimens covering diverse disease stages, multiple regions and varied demographic backgrounds. To overcome this barrier, the development of minimally invasive biopsy techniques, along with ethically sound and standardised sample acquisition protocols, is essential. These approaches enhance sample accessibility while reducing patient risk [94]. Additionally, conducting multicenter clinical cohort studies can expand sample diversity and representativeness. Establishing regional biobanks will facilitate the exchange of tissues and data between institutions, accelerating discovery and improving the reliability of findings [95]. (3) Sample heterogeneity and lack of standardised data processing. Sample variability—arising from factors such as patient age, sex, comorbidities, cardiac region (e.g., apex vs. base), and sampling conditions (e.g., cold ischemia time)—can impact transcriptomic consistency (Remoundou et al. [96]). Multicenter biobanks and open‐access databases promoting global collaboration are vital to constructing comprehensive single‐cell atlases of the human heart. Expanding the representation of various races, age groups and disease subtypes will enhance the generalizability and reproducibility of therapeutic targets [97]. (4) Limited cross‐platform integration. Differences in data formats, sequencing depth and cell type annotations across platforms such as 10× Genomics and Smart‐seq hinder integrative analyses and model generalisation. Promoting the integration of multiple omics—such as scRNA‐seq, spatial transcriptomics, scATAC‐seq and CITE‐seq—will facilitate the reconstruction of regulatory networks and spatial architectures. For instance, Litviňuková et al. [59] constructed a cellular atlas of the adult human heart, revealing regional differences in cellular composition and function. Differences in data formats, sequencing depth and cell type annotations across platforms such as 10× Genomics and Smart‐seq hinder integrative analyses and model generalisation. Promoting the integration of multiple omics—such as scRNA‐seq, spatial transcriptomics, scATAC‐seq, and CITE‐seq—will facilitate the reconstruction of regulatory networks and spatial architectures [98]. Machine learning approaches applied to single‐cell datasets have proven effective in predicting cell identity and phenotypic transitions, laying the foundation for robust translational models. Such cross‐species comparative frameworks will be essential for future drug development and personalised medicine.

## 5 Conclusions and Future Directions

**FIGURE 5:**
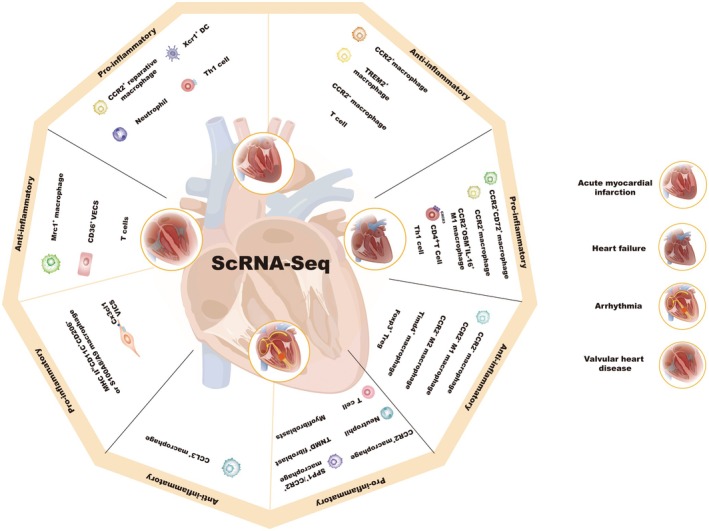
Utilising SCS technology, the heterogeneity of immune cell subsets in CDs is clarified. Different immune cell subsets play distinct roles in the onset and progression of various CDs. ScRNA‐seq, single‐cell RNA sequencing.

SCS is revolutionising our understanding of CDs by providing high‐resolution, unbiased insights into immune cell infiltration and their roles in disease progression. The technology enables the identification of immune cell heterogeneity at the single‐cell level, revealing rare cell types, functional differences and novel interactions that drive cardiac pathology [26]. In particular, macrophages exhibit significant plasticity and play complex roles in conditions such as MI, HF and arrhythmias. Recent advances in scRNA‐seq have highlighted the dynamic shifts in macrophage populations—pro‐inflammatory macrophages predominate early in disease, while reparative macrophages emerge in later stages [3]. Additionally, DCs and lymphocytes, though less studied, represent emerging targets for immunotherapy in cardiovascular diseases (Figure 5).

The emergence of scRNA‐seq has enabled researchers to construct high‐resolution immune cell atlases in CDs at single‐cell resolution, uncovering both shared immune mechanisms and disease‐specific response patterns under various pathological conditions. These discoveries have not only deepened our understanding of the immune system's role in cardiac pathophysiology but also laid a theoretical foundation for immune‐targeted interventions. Current studies have identified several conserved immune response features shared across different CDs, including the following: (1) Common dynamics in immune cell lineage evolution: Numerous studies have revealed that in acute AMI, HF, arrhythmia and VHD, the monocyte–macrophage lineage exhibits remarkable plasticity. During the early disease phase, M1‐like pro‐inflammatory macrophages are predominantly activated, followed by a gradual transition toward M2‐like reparative subpopulations [24, 99]. (2) Conserved neutrophil–macrophage activation axis: In the acute phase following cardiac injury, neutrophils rapidly infiltrate myocardial tissue, followed by the differentiation of resident or bone marrow–derived monocytes into various macrophage subtypes (e.g., TREM2^+^ and SPP1^+^). This transition has been consistently observed in multiple disease models, including AMI, HF and arrhythmias [24, 44]. (3) Shared upregulation of pro‐inflammatory cytokines with cell‐type specificity: Cytokines such as IL‐1β, TNF‐α, CCL2 and CXCL8 are broadly upregulated across various CDs and are predominantly produced by Ly6C^^hi^ monocytes and M1‐type macrophages. ScRNA‐seq has provided high‐resolution insights into the cellular origins of these cytokines, revealing subtype‐specific expression patterns that offer potential targets for anti‐inflammatory therapy [3].

Beyond these shared features, individual CDs exhibit distinct alterations in immune cell lineages, activation states and functional roles. These disease‐specific immune responses likely reflect adaptive regulatory mechanisms shaped by different pathophysiological microenvironments. We summarised these distinct immune features identified through scRNA‐seq in major cardiac conditions in Table 2, aiming to support the development of more precise immunomodulatory therapies. Moreover, the integration of emerging multi‐omics technologies—such as CITE‐seq, spatial transcriptomics and scATAC‐seq—has enabled comprehensive analysis of immune cell spatial localisation, epigenetic modifications and transcriptional regulatory networks under diverse cardiac disease conditions. These advances provide a powerful toolbox to dissect the context‐specific immune landscape of the heart [25, 101].

**TABLE 2:** Disease‐specific immune cell alterations in major cardiac pathologies revealed by scRNA‐seq and spatial omics.

Disease	Immune cell lineage alterations	Activation and functional role	Spatial distribution	Epigenetic/regulatory insights	References
Acute myocardial infarction (AMI)	Rapid recruitment of Ly6Chi monocytes; emergence of TREM2^+^ and SPP1^+^ macrophage subtypes	Early pro‐inflammatory macrophages (M1‐like); transition to reparative M2‐like macrophages secreting IL‐10, TGF‐β, VEGF	TREM2^+^ macrophages enriched in infarct core; SPP1^+^ cells at border zones	TREM2^+^ macrophages upregulated lipid metabolism and wound healing genes via PPAR signalling	[19, 24, 25]
Heart failure (HF)	Continuous infiltration of CCR2^+^ monocytes; expansion of CD72^+^ and Ccrl2^+^ inflammatory macrophages	Chronic MHC‐II^hi macrophages sustain low‐grade inflammation; promote cardiac fibrosis	Inflammatory macrophages enriched in failing myocardium and perivascular regions	Chromatin accessibility profiling reveals NF‐κB, IRF5 activation in HF‐associated macrophages	[42, 43, 45]
Arrhythmia (esp. AF)	SPP1^+^ M1‐like macrophage subtype increases; IL‐1β^+^ macrophage subset prevalent	Promote atrial fibrosis and electrical remodelling; induce ion channel reprogramming in cardiomyocytes	Preferential accumulation in fibrotic atrial regions (e.g., posterior wall and PV junction)	IL‐1β/STAT3 axis drives macrophage–cardiomyocyte crosstalk	[61, 88]
Valvular heart disease (VHD)	Aggregation of S100A8^+^/S100A9^+^ inflammatory macrophages; increased CD11c^+^MHCII^hi MΦ, reduced Mrc1^+^ anti‐inflammatory MΦ	Pro‐inflammatory macrophages promote valve thickening; anti‐inflammatory macrophages attempt local immune suppression	Infiltrated macrophages localised in subendothelial and calcified regions of aortic valves	TLR2/TLR4 signalling activation promotes monocyte‐to‐macrophage transition; possible NF‐κB involvement	[70, 71, 100]

With the rapid advancement of single‐cell omics technologies, the integration of multi‐omics platforms is reshaping our understanding of the molecular and cellular mechanisms underlying cardiovascular disease. These approaches offer a multidimensional view of critical immune and stromal cell populations, enabling the identification of regulatory networks that govern pathological remodelling. In AMI, the combination of scRNA‐seq and scATAC‐seq has revealed dynamic changes in both transcriptional profiles and chromatin accessibility of macrophages during the transition from inflammatory to reparative phases. This strategy identified key transcription factors such as IRF8 and SPI1, and reconstructed cell‐specific regulatory networks, providing epigenetic insights into immune cell fate decisions [25]. Concurrently, spatial transcriptomics has mapped the migratory trajectories of fibroblasts and immune cells from infarct cores to border zones, highlighting how spatial microenvironments influence cell state transitions. Multimodal single‐cell platforms such as CITE‐seq have further characterised tissue‐reparative macrophage subsets, linking their phenotypes to specific secretory profiles, including TGF‐β and IGF1 [102]. In studies of VHD, single‐cell transcriptomics has elucidated the differentiation trajectories of fibroblasts into RUNX2^+^ osteoblast‐like cells during aortic valve calcification, as well as the crosstalk between immune cells and osteogenic transformation [103]. Integrating scATAC‐seq has uncovered chromatin accessibility changes at key regulatory loci, offering novel insights into the epigenetic basis of calcification. In addition, research on BAV‐associated thoracic aortic aneurysm has identified disease‐specific chromatin remodelling events and fate shifts in vascular cells, revealing potential molecular targets for intervention [104]. Looking ahead, constructing spatiotemporal multi‐omic atlases that integrate spatial, transcriptomic, epigenomic and proteomic dimensions will be essential to deciphering the complex regulatory landscapes of cardiovascular pathology. Furthermore, combining cardiac organoid models with spatial omics holds promise for simulating human‐specific microenvironments, overcoming species‐related limitations of traditional animal models and advancing precision medicine strategies [26, 92]. Therefore, future directions should focus on the creation of cellular atlases with enhanced resolution, the incorporation of multi‐omics analyses, single‐cell dynamic monitoring and spatial–temporal analysis [16]. These advancements will provide deeper insights into immune cell functions and regulatory pathways, facilitating the development of targeted immunotherapies [26]. Moreover, enhancing methods for tissue collection and preservation will be crucial for translating these findings into clinical applications.

In conclusion, the integration of SCS with multidisciplinary approaches holds great potential for uncovering novel biomarkers, therapeutic targets and precision treatments for CDs. Collaborative efforts between basic scientists, clinicians and engineers are essential to overcome existing challenges and harness the full potential of this transformative technology for improving patient outcomes.

## Author Contributions

**Weirong Zeng:** data curation (equal), writing – original draft (equal). **Jiao Li:** writing – original draft (equal). **Weiwei Liu:** data curation (equal). **Ranzun Zhao:** project administration (equal). **Bei Shi:** supervision (equal), writing – review and editing (equal). **Yan Wang:** writing – review and editing (equal).

## Conflicts of Interest

The authors declare no conflicts of interest.

## Data Availability

No new data were generated or analysed in this study. Data sharing is not applicable to this article.

## References

[1] H. Thomas , J. Diamond , A. Vieco , et al., “Global Atlas of Cardiovascular Disease 2000‐2016: The Path to Prevention and Control,” Global Heart 13 (2018): 143–163. 30301680 10.1016/j.gheart.2018.09.511

[2] D. Zhao , J. Liu , M. Wang , X. Zhang , and M. Zhou , “Epidemiology of Cardiovascular Disease in China: Current Features and Implications,” Nature Reviews. Cardiology 16 (2019): 203–212. 30467329 10.1038/s41569-018-0119-4

[3] F. K. Swirski and M. Nahrendorf , “Cardioimmunology: The Immune System in Cardiac Homeostasis and Disease,” Nature Reviews. Immunology 18 (2018): 733–744. 10.1038/s41577-018-0065-8 30228378

[4] C. Peet , A. Ivetic , D. I. Bromage , and A. M. Shah , “Cardiac Monocytes and Macrophages After Myocardial Infarction,” Cardiovascular Research 116 (2020): 1101–1112. 31841135 10.1093/cvr/cvz336 PMC7177720

[5] E. Mass , F. Nimmerjahn , K. Kierdorf , and A. Schlitzer , “Tissue‐Specific Macrophages: How They Develop and Choreograph Tissue Biology,” Nature Reviews. Immunology 23 (2023): 563–579. 10.1038/s41577-023-00848-y PMC10017071 36922638

[6] T. Lazarov , S. Juarez‐Carreño , N. Cox , and F. Geissmann , “Physiology and Diseases of Tissue‐Resident Macrophages,” Nature 618 (2023): 698–707. 37344646 10.1038/s41586-023-06002-x PMC10649266

[7] J.‐H. Choi , Y. do , C. Cheong , et al., “Identification of Antigen‐Presenting Dendritic Cells in Mouse Aorta and Cardiac Valves,” Journal of Experimental Medicine 206 (2009): 497–505. 19221394 10.1084/jem.20082129 PMC2699134

[8] J. Barbi , D. Pardoll , and F. Pan , “Treg Functional Stability and Its Responsiveness to the Microenvironment,” Immunological Reviews 259 (2014): 115–139. 24712463 10.1111/imr.12172 PMC3996455

[9] J. W. Williams , C. Giannarelli , A. Rahman , G. J. Randolph , and J. C. Kovacic , “Macrophage Biology, Classification, and Phenotype in Cardiovascular Disease,” Journal of the American College of Cardiology 72 (2018): 2166–2180. 30360826 10.1016/j.jacc.2018.08.2148 PMC6209330

[10] Y. Chen , Y. Liu , and X. Gao , “The Application of Single‐Cell Technologies in Cardiovascular Research,” Frontiers in Cell and Development Biology 9 (2021): 751371. 10.3389/fcell.2021.751371 PMC8542723 34708045

[11] X. Xu , X. Hua , H. Mo , S. Hu , and J. Song , “Single‐Cell RNA Sequencing to Identify Cellular Heterogeneity and Targets in Cardiovascular Diseases: From Bench to Bedside,” Basic Research in Cardiology 118 (2023): 7. 36750503 10.1007/s00395-022-00972-1

[12] Y. Fan , H. Zhou , X. Liu , et al., “Applications of Single‐Cell RNA Sequencing in Cardiovascular Research,” Frontiers in Cell and Developmental Biology 9 (2021): 810232. 35174168 10.3389/fcell.2021.810232 PMC8841340

[13] E. Bianconi , A. Piovesan , F. Facchin , et al., “An Estimation of the Number of Cells in the Human Body,” Annals of Human Biology 40 (2013): 463–471. 23829164 10.3109/03014460.2013.807878

[14] A. Eldar and M. B. Elowitz , “Functional Roles for Noise in Genetic Circuits,” Nature 467 (2010): 167–173. 20829787 10.1038/nature09326 PMC4100692

[15] A. K. Shalek , R. Satija , J. Shuga , et al., “Single‐Cell RNA‐Seq Reveals Dynamic Paracrine Control of Cellular Variation,” Nature 510 (2014): 363–369. 24919153 10.1038/nature13437 PMC4193940

[16] S. S. Potter , “Single‐Cell RNA Sequencing for the Study of Development, Physiology and Disease,” Nature Reviews. Nephrology 14 (2018): 479–492. 29789704 10.1038/s41581-018-0021-7 PMC6070143

[17] C. Ziegenhain , B. Vieth , S. Parekh , et al., “Comparative Analysis of Single‐Cell RNA Sequencing Methods,” Molecular Cell 65 (2017): 631–643.e4. 28212749 10.1016/j.molcel.2017.01.023

[18] D. Jovic , X. Liang , H. Zeng , L. Lin , F. Xu , and Y. Luo , “Single‐Cell RNA Sequencing Technologies and Applications: A Brief Overview,” Clinical and Translational Medicine 12 (2022): e694. 35352511 10.1002/ctm2.694 PMC8964935

[19] C. Cochain , E. Vafadarnejad , P. Arampatzi , et al., “Single‐Cell RNA‐Seq Reveals the Transcriptional Landscape and Heterogeneity of Aortic Macrophages in Murine Atherosclerosis,” Circulation Research 122 (2018): 1661–1674. 29545365 10.1161/CIRCRESAHA.117.312509

[20] S. Picelli , Å. K. Björklund , O. R. Faridani , S. Sagasser , G. Winberg , and R. Sandberg , “Smart‐Seq2 for Sensitive Full‐Length Transcriptome Profiling in Single Cells,” Nature Methods 10 (2013): 1096–1098. 24056875 10.1038/nmeth.2639

[21] D. A. Skelly , G. T. Squiers , M. A. McLellan , et al., “Single‐Cell Transcriptional Profiling Reveals Cellular Diversity and Intercommunication in the Mouse Heart,” Cell Reports 22 (2018): 600–610. 29346760 10.1016/j.celrep.2017.12.072

[22] M. Wu , M. Xia , W. Li , and H. Li , “Single‐Cell Sequencing Applications in the Inner Ear,” Frontiers in Cell and Development Biology 9 (2021): 637779. 10.3389/fcell.2021.637779 PMC7907461 33644075

[23] J. Ding , X. Adiconis , S. K. Simmons , et al., “Systematic Comparison of Single‐Cell and Single‐Nucleus RNA‐Sequencing Methods,” Nature Biotechnology 38 (2020): 737–746. 10.1038/s41587-020-0465-8 PMC7289686 32341560

[24] S.‐H. Jung , B. H. Hwang , S. Shin , et al., “Spatiotemporal Dynamics of Macrophage Heterogeneity and a Potential Function of Trem2hi Macrophages in Infarcted Hearts,” Nature Communications 13 (2022): 4580. 10.1038/s41467-022-32284-2 PMC9357004 35933399

[25] C. Kuppe , R. O. Ramirez Flores , Z. Li , et al., “Spatial Multi‐Omic Map of Human Myocardial Infarction,” Nature 608 (2022): 766–777. 35948637 10.1038/s41586-022-05060-x PMC9364862

[26] D. T. Paik , S. Cho , L. Tian , H. Y. Chang , and J. C. Wu , “Single‐Cell RNA Sequencing in Cardiovascular Development, Disease and Medicine,” Nature Reviews. Cardiology 17 (2020): 457–473. 32231331 10.1038/s41569-020-0359-y PMC7528042

[27] M. Ackers‐Johnson , W. L. W. Tan , and R. S.‐Y. Foo , “Following Hearts, One Cell at a Time: Recent Applications of Single‐Cell RNA Sequencing to the Understanding of Heart Disease,” Nature Communications 9 (2018): 4434. 10.1038/s41467-018-06894-8 PMC6207674 30375391

[28] K. Jin , S. Gao , P. Yang , et al., “Single‐Cell RNA Sequencing Reveals the Temporal Diversity and Dynamics of Cardiac Immunity After Myocardial Infarction,” Small Methods 6 (2022): e2100752. 35023642 10.1002/smtd.202100752

[29] S. Gong , M. Zhai , J. Shi , et al., “TREM2 Macrophage Promotes Cardiac Repair in Myocardial Infarction by Reprogramming Metabolism via SLC25A53,” Cell Death and Differentiation 31 (2024): 239–253, 10.1038/s41418-023-01252-8. 38182899 PMC10850484

[30] E. Wan , X. Y. Yeap , S. Dehn , et al., “Enhanced Efferocytosis of Apoptotic Cardiomyocytes Through Myeloid‐Epithelial‐Reproductive Tyrosine Kinase Links Acute Inflammation Resolution to Cardiac Repair After Infarction,” Circulation Research 113 (2013): 1004–1012. 23836795 10.1161/CIRCRESAHA.113.301198 PMC3840464

[31] M. Horckmans , L. Ring , J. Duchene , et al., “Neutrophils Orchestrate Post‐Myocardial Infarction Healing by Polarizing Macrophages Towards a Reparative Phenotype,” European Heart Journal 38 (2017): 187–197. 28158426 10.1093/eurheartj/ehw002

[32] L. Zhuang , L. Lu , R. Zhang , K. Chen , and X. Yan , “Comprehensive Integration of Single‐Cell Transcriptional Profiling Reveals the Heterogeneities of Non‐Cardiomyocytes in Healthy and Ischemic Hearts,” Frontiers in Cardiovascular Medicine 7 (2020): 615161. 33365332 10.3389/fcvm.2020.615161 PMC7750309

[33] Y. Xu , K. Jiang , F. Su , et al., “A Transient Wave of Bhlhe41+ Resident Macrophages Enables Remodeling of the Developing Infarcted Myocardium,” Cell Reports 42 (2023): 113174. 37751357 10.1016/j.celrep.2023.113174

[34] Y. Liu , Y. H. Shao , J. M. Zhang , et al., “Macrophage CARD9 Mediates Cardiac Injury Following Myocardial Infarction Through Regulation of Lipocalin 2 Expression,” Signal Transduction and Targeted Therapy 8 (2023): 394. 37828006 10.1038/s41392-023-01635-w PMC10570328

[35] H. Inui , M. Nishida , M. Ichii , et al., “Xcr1^+^ Conventional Dendritic Cell‐Induced CD4^+^ T Helper 1 Cell Activation Exacerbates Cardiac Remodeling After Ischemic Myocardial Injury,” Journal of Molecular and Cellular Cardiology 176 (2023): 68–83. 36739942 10.1016/j.yjmcc.2023.01.011

[36] U. Hofmann and S. Frantz , “Role of T‐Cells in Myocardial Infarction,” European Heart Journal 37 (2016): 873–879. 26646702 10.1093/eurheartj/ehv639

[37] Y. Xia , D. Gao , X. Wang , et al., “Role of Treg Cell Subsets in Cardiovascular Disease Pathogenesis and Potential Therapeutic Targets,” Frontiers in Immunology 15 (2024): 1331609. 38558816 10.3389/fimmu.2024.1331609 PMC10978666

[38] G. V. Halade and D. H. Lee , “Inflammation and Resolution Signaling in Cardiac Repair and Heart Failure,” eBioMedicine 79 (2022): 103992. 35405389 10.1016/j.ebiom.2022.103992 PMC9014358

[39] W. Liu , Y. Li , Y. Zhang , et al., “Identification of Biomarkers and Immune Infiltration in Acute Myocardial Infarction and Heart Failure by Integrated Analysis,” Bioscience Reports 43 (2023): BSR20222552. 37334672 10.1042/BSR20222552 PMC10329185

[40] C. Panico , M. Kallikourdis , and G. Condorelli , “Defining Circulating Mononuclear Cells in Heart Failure Through Single‐Cell RNA Sequencing: New Insights for an Old Disease,” Cardiovascular Research 117 (2021): 341–342. 33049776 10.1093/cvr/cvaa156

[41] E. Martini , P. Kunderfranco , C. Peano , et al., “Single‐Cell Sequencing of Mouse Heart Immune Infiltrate in Pressure Overload‐Driven Heart Failure Reveals Extent of Immune Activation,” Circulation 140 (2019): 2089–2107. 31661975 10.1161/CIRCULATIONAHA.119.041694

[42] S. A. Dick , J. A. Macklin , S. Nejat , et al., “Self‐Renewing Resident Cardiac Macrophages Limit Adverse Remodeling Following Myocardial Infarction,” Nature Immunology 20 (2019): 29–39. 30538339 10.1038/s41590-018-0272-2 PMC6565365

[43] C. Panico , A. Felicetta , P. Kunderfranco , et al., “Single‐Cell RNA Sequencing Reveals Metabolic Stress‐Dependent Activation of Cardiac Macrophages in a Model of Dyslipidemia‐Induced Diastolic Dysfunction,” Circulation 150 (2023): e062984, 10.1161/CIRCULATIONAHA.122.062984. 38126199

[44] N. R. Wong , J. Mohan , B. J. Kopecky , et al., “Resident Cardiac Macrophages Mediate Adaptive Myocardial Remodeling,” Immunity 54 (2021): 2072–2088.e7. 34320366 10.1016/j.immuni.2021.07.003 PMC8446343

[45] S.‐H. Ni , J. D. Xu , S. N. Sun , et al., “Single‐Cell Transcriptomic Analyses of Cardiac Immune Cells Reveal That Rel‐Driven CD72‐Positive Macrophages Induce Cardiomyocyte Injury,” Cardiovascular Research 118 (2022): 1303–1320. 34100920 10.1093/cvr/cvab193

[46] L. Zhuang , X. Zong , Q. Yang , Q. Fan , and R. Tao , “Interleukin‐34‐NF‐κB Signaling Aggravates Myocardial Ischemic/Reperfusion Injury by Facilitating Macrophage Recruitment and Polarization,” eBioMedicine 95 (2023): 104744. 37556943 10.1016/j.ebiom.2023.104744 PMC10433018

[47] D. Ramanujam , A. P. Schön , C. Beck , et al., “MicroRNA‐21‐Dependent Macrophage‐To‐Fibroblast Signaling Determines the Cardiac Response to Pressure Overload,” Circulation 143 (2021): 1513–1525. 33550817 10.1161/CIRCULATIONAHA.120.050682 PMC8032214

[48] F. Laroumanie , V. Douin‐Echinard , J. Pozzo , et al., “CD4^+^ T Cells Promote the Transition From Hypertrophy to Heart Failure During Chronic Pressure Overload,” Circulation 129 (2014): 2111–2124. 24657994 10.1161/CIRCULATIONAHA.113.007101

[49] S.‐L. Chang , Y. W. Hsiao , Y. N. Tsai , et al., “Interleukin‐17 Enhances Cardiac Ventricular Remodeling via Activating MAPK Pathway in Ischemic Heart Failure,” Journal of Molecular and Cellular Cardiology 122 (2018): 69–79. 30096409 10.1016/j.yjmcc.2018.08.005

[50] S. Finger , M. Knorr , M. Molitor , et al., “A Sequential Interferon Gamma Directed Chemotactic Cellular Immune Response Determines Survival and Cardiac Function Post‐Myocardial Infarction,” Cardiovascular Research 115 (2019): 1907–1917. 30949687 10.1093/cvr/cvz092

[51] E. Azizi , A. J. Carr , G. Plitas , et al., “Single‐Cell Map of Diverse Immune Phenotypes in the Breast Tumor Microenvironment,” Cell 174 (2018): 1293–1308.e36. 29961579 10.1016/j.cell.2018.05.060 PMC6348010

[52] L. Li , Q. Ma , M. Wang , et al., “Single‐Cell Transcriptome Sequencing of Macrophages in Common Cardiovascular Diseases,” Journal of Leukocyte Biology 113 (2023): 139–148. 36822177 10.1093/jleuko/qiac014

[53] C. Rischpler , T. Rassaf , L. Umutlu , K. Herrmann , T. W. Schlosser , and M. Totzeck , “Imaging the Inflammatory Response in Checkpoint Inhibition Myocarditis,” Journal of Nuclear Medicine 63 (2022): 14–16. 34857662 10.2967/jnumed.121.262301 PMC8717203

[54] N. Ngwenyama , A. M. Salvador , F. Velázquez , et al., “CXCR3 Regulates CD4^+^ T Cell Cardiotropism in Pressure Overload‐Induced Cardiac Dysfunction,” JCI Insight 4 (2019): e125527. 30779709 10.1172/jci.insight.125527 PMC6483643

[55] S. E. Choi , D. Sagris , A. Hill , G. Y. H. Lip , and A. H. Abdul‐Rahim , “Atrial Fibrillation and Stroke,” Expert Review of Cardiovascular Therapy 21 (2023): 35–56. 36537565 10.1080/14779072.2023.2160319

[56] Y. Yao , M. Yang , D. Liu , and Q. Zhao , “Immune Remodeling and Atrial Fibrillation,” Frontiers in Physiology 13 (2022): 927221. 35936905 10.3389/fphys.2022.927221 PMC9355726

[57] S. Poelzing and D. S. Rosenbaum , “Altered Connexin43 Expression Produces Arrhythmia Substrate in Heart Failure,” American Journal of Physiology. Heart and Circulatory Physiology 287 (2004): H1762–H1770. 15205174 10.1152/ajpheart.00346.2004

[58] E. Dupont , T. Matsushita , R. A. Kaba , et al., “Altered Connexin Expression in Human Congestive Heart Failure,” Journal of Molecular and Cellular Cardiology 33 (2001): 359–371. 11162139 10.1006/jmcc.2000.1308

[59] M. Litviňuková , “Cells of the Adult Human Heart,” Nature 588 (2020): 466–472. 32971526 10.1038/s41586-020-2797-4 PMC7681775

[60] J. Sugita , K. Fujiu , Y. Nakayama , et al., “Cardiac Macrophages Prevent Sudden Death During Heart Stress,” Nature Communications 12 (2021): 1910. 10.1038/s41467-021-22178-0 PMC7997915 33771995

[61] M. Hulsmans , “Recruited Macrophages Elicit Atrial Fibrillation,” Science 381 (2023): 231–239. 37440641 10.1126/science.abq3061 PMC10448807

[62] Y. Wu , S. Zhan , L. Chen , et al., “TNFSF14/LIGHT Promotes Cardiac Fibrosis and Atrial Fibrillation Vulnerability via PI3Kγ/SGK1 Pathway‐Dependent M2 Macrophage Polarisation,” Journal of Translational Medicine 21 (2023): 544. 37580750 10.1186/s12967-023-04381-3 PMC10424430

[63] M. Fu , X. Hua , S. Shu , et al., “Single‐Cell RNA Sequencing in Donor and End‐Stage Heart Failure Patients Identifies NLRP3 as a Therapeutic Target for Arrhythmogenic Right Ventricular Cardiomyopathy,” BMC Medicine 22 (2024): 11. 38185631 10.1186/s12916-023-03232-8 PMC10773142

[64] B. Iung and A. Vahanian , “Epidemiology of Acquired Valvular Heart Disease,” Canadian Journal of Cardiology 30 (2014): 962–970. 24986049 10.1016/j.cjca.2014.03.022

[65] A. Hulin , L. Hortells , M. V. Gomez‐Stallons , et al., “Maturation of Heart Valve Cell Populations During Postnatal Remodeling,” Development 146 (2019): dev173047. 30796046 10.1242/dev.173047 PMC6602342

[66] A. Shigeta , V. Huang , J. Zuo , et al., “Endocardially Derived Macrophages Are Essential for Valvular Remodeling,” Developmental Cell 48 (2019): 617–630.e3. 30799229 10.1016/j.devcel.2019.01.021 PMC6440481

[67] C. Y. Y. Yip and C. A. Simmons , “The Aortic Valve Microenvironment and Its Role in Calcific Aortic Valve Disease,” Cardiovascular Pathology 20 (2011): 177–182. 21256052 10.1016/j.carpath.2010.12.001

[68] J. C. Grim , B. A. Aguado , B. J. Vogt , et al., “Secreted Factors From Proinflammatory Macrophages Promote an Osteoblast‐Like Phenotype in Valvular Interstitial Cells,” Arteriosclerosis, Thrombosis, and Vascular Biology 40 (2020): e296–e308. 32938214 10.1161/ATVBAHA.120.315261 PMC7578003

[69] P. R. Goody , M. R. Hosen , D. Christmann , et al., “Aortic Valve Stenosis: From Basic Mechanisms to Novel Therapeutic Targets,” Arteriosclerosis, Thrombosis, and Vascular Biology 40 (2020): 885–900. 32160774 10.1161/ATVBAHA.119.313067

[70] K. Wang , Q. Zheng , X. Liu , B. C. Geng , N. G. Dong , and J. W. Shi , “Identifying Hub Genes of Calcific Aortic Valve Disease and Revealing the Immune Infiltration Landscape Based on Multiple WGCNA and Single‐Cell Sequence Analysis,” Frontiers in Immunology 13 (2022): 1035285. 36405745 10.3389/fimmu.2022.1035285 PMC9673246

[71] S. H. Lee , N. Kim , M. Kim , et al., “Single‐Cell Transcriptomics Reveal Cellular Diversity of Aortic Valve and the Immunomodulation by PPARγ During Hyperlipidemia,” Nature Communications 13 (2022): 5461. 10.1038/s41467-022-33202-2 PMC9482653 36115863

[72] F. Iqbal , F. Schlotter , D. Becker‐Greene , et al., “Sortilin Enhances Fibrosis and Calcification in Aortic Valve Disease by Inducing Interstitial Cell Heterogeneity,” European Heart Journal 44 (2023): 885–898. 36660854 10.1093/eurheartj/ehac818 PMC9991042

[73] L.‐H. M. Moncla , M. Briend , Y. Bossé , and P. Mathieu , “Calcific Aortic Valve Disease: Mechanisms, Prevention and Treatment,” Nature Reviews. Cardiology 20 (2023): 546–559. 36829083 10.1038/s41569-023-00845-7

[74] T. Lyu , Y. Liu , B. Li , R. Xu , J. Guo , and D. Zhu , “Single‐Cell Transcriptomics Reveals Cellular Heterogeneity and Macrophage‐To‐Mesenchymal Transition in Bicuspid Calcific Aortic Valve Disease,” Biology Direct 18 (2023): 35. 37391760 10.1186/s13062-023-00390-w PMC10311753

[75] T. Cartwright , N. D. Perkins , and L. Wilson , “NFKB1: A Suppressor of Inflammation, Ageing and Cancer,” FEBS Journal 283 (2016): 1812–1822. 26663363 10.1111/febs.13627

[76] S. P. Souza , S. D. Splitt , J. C. Sànchez‐Arcila , et al., “Genetic Mapping Reveals Nfkbid as a Central Regulator of Humoral Immunity to Toxoplasma Gondii,” PLoS Pathogens 17 (2021): e1010081. 34871323 10.1371/journal.ppat.1010081 PMC8675933

[77] Y. Zhang , J. Tu , Y. Li , et al., “Inflammation Macrophages Contribute to Cardiac Homeostasis,” Cardiology Plus 8, no. 1 (2023): 6–17.

[78] Y. Shen , I.‐M. Kim , N. L. Weintraub , and Y. Tang , “Identification of the Metabolic State of Surviving Cardiomyocytes in the Human Infarcted Heart by Spatial Single‐Cell Transcriptomics,” Cardiology Plus 8 (2023): 18–26. 37187809 10.1097/CP9.0000000000000038 PMC10180026

[79] Z. Huang and A. Sun , “Metabolism, Inflammation, and Cardiovascular Diseases From Basic Research to Clinical Practice,” Cardiology Plus 8 (2023): 4–5. 37206089 10.1097/CP9.0000000000000037 PMC10178914

[80] Z. Ren , P. Yu , D. Li , et al., “Single‐Cell Reconstruction of Progression Trajectory Reveals Intervention Principles in Pathological Cardiac Hypertrophy,” Circulation 141 (2020): 1704–1719. 32098504 10.1161/CIRCULATIONAHA.119.043053

[81] M. C. Hill , B. Simonson , C. Roselli , et al., “Large‐Scale Single‐Nuclei Profiling Identifies Role for ATRNL1 in Atrial Fibrillation,” Nature Communications 15 (2024): 10002. 10.1038/s41467-024-54296-w PMC11576987 39562555

[82] G. Bajpai , C. Schneider , N. Wong , et al., “The Human Heart Contains Distinct Macrophage Subsets With Divergent Origins and Functions,” Nature Medicine 24 (2018): 1234–1245. 10.1038/s41591-018-0059-x PMC6082687 29892064

[83] Z. Ma , M. Li , R. Guo , et al., “Treating Myocardial Infarction via a Nano‐Ultrasonic Contrast Agent‐Mediated High‐Efficiency Drug Delivery System Targeting Macrophages,” Science Advances 11 (2025): eadp7126. 39752485 10.1126/sciadv.adp7126 PMC11698097

[84] M. Fu , S. Jia , L. Xu , et al., “Single‐Cell Multiomic Analysis Identifies Macrophage Subpopulations in Promoting Cardiac Repair,” Journal of Clinical Investigation 134 (2024): e175297. 39190625 10.1172/JCI175297 PMC11444165

[85] Q. Wu , Q. Yao , T. Hu , et al., “Dapagliflozin Protects Against Chronic Heart Failure in Mice by Inhibiting Macrophage‐Mediated Inflammation, Independent of SGLT2,” Cell Reports Medicine 4 (2023): 101334. 38118414 10.1016/j.xcrm.2023.101334 PMC10772464

[86] G. Li , C. Zhang , Y. Li , et al., “Optogenetic Vagal Nerve Stimulation Attenuates Heart Failure by Limiting the Generation of Monocyte‐Derived Inflammatory CCRL2+ Macrophages,” Immunity 58 (2025): 1847–1861.e9. 40580954 10.1016/j.immuni.2025.06.003

[87] T. Mesquita and E. Cingolani , “Targeting Arrhythmogenic Macrophages: Lessons Learned From Arrhythmogenic Cardiomyopathy,” Journal of Clinical Investigation 134 (2024): e180482. 38747296 10.1172/JCI180482 PMC11093592

[88] Y. Suzuki , T. Emoto , S. Sato , et al., “Left Atrial Single‐Cell Transcriptomics Reveals Amphiregulin as a Surrogate Marker for Atrial Fibrillation,” Communications Biology 7 (2024): 1601. 39622943 10.1038/s42003-024-07308-w PMC11612213

[89] L. A. Meier , J. L. Auger , B. J. Engelson , et al., “CD301b/MGL2^+^ Mononuclear Phagocytes Orchestrate Autoimmune Cardiac Valve Inflammation and Fibrosis,” Circulation 137 (2018): 2478–2493. 29386201 10.1161/CIRCULATIONAHA.117.033144 PMC5988921

[90] M. Qiu , J. B. Zong , Q. W. He , et al., “Cell Heterogeneity Uncovered by Single‐Cell RNA Sequencing Offers Potential Therapeutic Targets for Ischemic Stroke,” Aging and Disease 13 (2022): 1436–1454. 36186129 10.14336/AD.2022.0212 PMC9466965

[91] F. Klimm , E. M. Toledo , T. Monfeuga , F. Zhang , C. M. Deane , and G. Reinert , “Functional Module Detection Through Integration of Single‐Cell RNA Sequencing Data With Protein‐Protein Interaction Networks,” BMC Genomics 21 (2020): 756. 33138772 10.1186/s12864-020-07144-2 PMC7607865

[92] L. Drakhlis , S. Biswanath , C. M. Farr , et al., “Human Heart‐Forming Organoids Recapitulate Early Heart and Foregut Development,” Nature Biotechnology 39 (2021): 737–746. 10.1038/s41587-021-00815-9 PMC8192303 33558697

[93] J. Young , J. Inamo , Z. Caterer , R. Krishna , and F. Zhang , “CellPhenoX: An eXplainable Cell‐Specific Machine Learning Method to Predict Clinical Phenotypes Using Single‐Cell Multi‐Omics,” bioRxiv, (2025), 10.1101/2025.01.24.634132. PMC12622556 40985689

[94] F. Remes Lenicov and N. E. Fink , “Ethical Issues in the Use of Leftover Samples and Associated Personal Data Obtained From Diagnostic Laboratories,” Clinica Chimica Acta 548 (2023): 117442. 10.1016/j.cca.2023.117442 PMC10257511 37308048

[95] S. Prince , S.‐N. Then , and K.‐A. O'Grady , “Determining the State of Guidance on Pediatric Biobanking for Researchers, HRECS, and Families: Regulatory Mapping of International Guidance,” European Journal of Pediatrics 183 (2024): 2477–2490. 38478133 10.1007/s00431-024-05469-8 PMC11035456

[96] K. Remoundou , T. Alexakis , N. Peppes , K. Demestichas , and E. Adamopoulou , “A Quality Control Methodology for Heterogeneous Vehicular Data Streams,” Sensors (Basel) 22 (2022): 1550. 35214486 10.3390/s22041550 PMC8877783

[97] M. Asp , S. Giacomello , L. Larsson , et al., “A Spatiotemporal Organ‐Wide Gene Expression and Cell Atlas of the Developing Human Heart,” Cell 179 (2019): 1647–1660.e19. 31835037 10.1016/j.cell.2019.11.025

[98] Q. Shi , X. Li , Q. Peng , C. Zhang , and L. Chen , “scDA: Single Cell Discriminant Analysis for Single‐Cell RNA Sequencing Data,” Computational and Structural Biotechnology Journal 19 (2021): 3234–3244. 34141142 10.1016/j.csbj.2021.05.046 PMC8187165

[99] X. Shi , L. Zhang , Y. Li , et al., “Integrative Analysis of Bulk and Single‐Cell RNA Sequencing Data Reveals Cell Types Involved in Heart Failure,” Frontiers in Bioengineering and Biotechnology 9 (2021): 779225. 35071201 10.3389/fbioe.2021.779225 PMC8766768

[100] J. Li , W. Qiao , Y. Liu , et al., “Constructing Immunomodulator Biosynthesis Factory in Grafting‐From DNA Hydrogel for Heart Valve Regeneration,” Advanced Materials 37 (2025): e2506728, 10.1002/adma.202506728. 40509588

[101] Z. Tang , F. Alrumaihi , W. M. Alwanian , et al., “The Future of Cardiology: Integrating Single‐Cell Transcriptomics With Multi‐Omics for Enhanced Cardiac Disease Insights,” Current Problems in Cardiology 50 (2025): 103005. 39894239 10.1016/j.cpcardiol.2025.103005

[102] J. M. Amrute , X. Luo , V. Penna , et al., “Targeting Immune‐Fibroblast Cell Communication in Heart Failure,” Nature 635 (2024): 423–433. 39443792 10.1038/s41586-024-08008-5 PMC12334188

[103] Y. Huang , C. Wang , T. Zhou , et al., “Lumican Promotes Calcific Aortic Valve Disease Through H3 Histone Lactylation,” European Heart Journal 45 (2024): 3871–3885. 38976370 10.1093/eurheartj/ehae407

[104] X.‐W. Liu , P. Wang , L. Zhang , et al., “Single‐Cell RNA Sequencing and ATAC Sequencing Identify Novel Biomarkers for Bicuspid Aortic Valve‐Associated Thoracic Aortic Aneurysm,” Frontiers in Cardiovascular Medicine 11 (2024): 1265378. 38685981 10.3389/fcvm.2024.1265378 PMC11057375

